# Clinical characteristics of acute pancreatitis in children: a single-center experience in Western China

**DOI:** 10.1186/s12876-021-01706-8

**Published:** 2021-03-09

**Authors:** Rui Zhong, Shali Tan, Yan Peng, Huan Xu, Xin Jiang, Yongfeng Yan, Muhan Lv, Li Liu, Xiaowei Tang

**Affiliations:** 1 Department of Gastroenterology, The Affiliated Hospital of Southwest Medical University, Luzhou, 646000 China; 2 Department of Digestive Endoscopy, The First Affiliated Hospital With Nanjing Medical University, Nanjing, China; 3 Department of General Surgery, The First Affiliated Hospital With Nanjing Medical University, Nanjing, China

**Keywords:** Acute pancreatitis, Acute recurrent pancreatitis, Child, Etiology

## Abstract

**Background:**

The diagnosis of pediatric pancreatitis has been increasing over the last 20 years. We aimed to compare the clinical characteristics for pediatric acute pancreatitis (AP) with adult AP, and investigate the risk factor for acute recurrent pancreatitis (ARP) in children.

**Method:**

From June 2013 to June 2019, a total of 130 pediatric patients with AP at the inpatient database were enrolled. Univariate analysis and multivariate Cox regression analysis were performed to identify the risk factors for ARP in children.

**Result:**

Major etiologic factors in 130 patients were biliary (31.5%), idiopathic (28.5%). The etiology of pancreatitis in children was markedly different from that in adults (*p* < 0.001). Compared with the adult patients, the pediatric patients had significantly lower severity (*p* = 0.018) and occurrence rate of pancreatic necrosis (*p* = 0.041), SIRS (*p* = 0.021), acute peripancreatic fluid collection (*p* = 0.014). Univariate and Multivariate Cox regression analysis showed that female (*p* = 0.020; OR 3.821; 95% CI 1.231–11.861), hypertriglyceridemia (*p* = 0.045; OR 3.111; 95% CI 1.024–9.447), pancreatic necrosis (*p* = 0.023; OR 5.768; 95% CI 1.278–26.034) were the independent risk factors of ARP. Hypertriglyceridemia AP had the highest risk of recurrence compared to other etiology (*p* = 0.035).

**Conclusion:**

Biliary and idiopathic disease were the major etiologies of AP in children. Children have simpler conditions than adults. Female, hypertriglyceridemia, and pancreatic necrosis were associated with the onset of ARP.

## Background

Acute pancreatitis (AP) is an inflammatory reaction that causes the digestion, edema, bleeding, and necrosis of pancreatic tissue after activation of various trypsin enzymes in pancreatic tissue, caused by a variety of etiologies [1]. Clinically, it is characterized by acute epigastric pain, vomiting, and elevated pancreatic amylase. It has been reported that the incidence of AP in children has increased in the past 20 years [2, 3]. At present, AP is more common in children over five years old, with an incidence of 3/100 000, but its severity is similar in children of all ages [3, 4]. The overall case fatality rate is less than 5% [2, 5]. Compared with adults, there are significant differences in the incidence, etiology, clinical manifestation, and prognosis of AP in children [6]. Therefore, the purpose of this study was to analyze the demographic characteristics, etiology, clinical manifestations, and prognosis of AP, compare to adult AP, and to investigate the risk factors for acute recurrent pancreatitis (ARP) in children in southwest China.

## Methods

### Ethical approval

This study was approved by the Clinical trial Ethics Committee of the affiliated Hospital of Southwest Medical University (batch number: KY2019054). Informed consent was waived.

### Study population

We collected data retrospectively on 130 children with first AP attack from June 2013 to June 2019 at the electronic inpatient database, while patients with relapse of AP or chronic pancreatitis were excluded. The diagnostic criteria for pediatric AP were in accordance with the guidelines of the International Pediatric Pancreatitis Study Group, the European Pancreatic Club, and the Hungarian Pancreatic Group [2, 7, 8]. Pediatric AP can be recognized in patients less than 18 years old, when two of the following three criteria are fulfilled: (1) abdominal pain compatible with AP, (2) serum amylase and/or lipase values ≥ 3 times the upper limits of normal, (3) imaging findings consistent with AP. The clinical classification of AP includes: mild acute pancreatitis (MAP), characterized by the absence of organ failure and local or systemic complications; moderately-severe acute pancreatitis (MSAP), characterized by transient organ failure (< 48 h), or accompanied by local or systemic complications; severe acute pancreatitis (SAP), characterized by persistent organ failure (> 48 h). The local complications include acute peripancreatic fluid collection (APFC), acute necrotic collection (ANC), and pancreatic pseudocyst (PPC). The systemic complications include organ failure, systemic inflammatory response syndrome (SIRS). The organ failure include acute respiratory distress syndrome (ARDS), shock, and acute renal failure (ARF). In addition, acute recurrent pancreatitis (ARP) is characterized by: Requires at least 2 distinct episodes of AP (each as defined above), along with: Complete resolution of pain (≥ 1-month pain-free interval between the diagnoses of AP) or complete normalization of serum pancreatic enzyme levels (amylase and lipase), before the subsequent episode of AP is diagnosed, along with complete resolution of pain symptoms, irrespective of a specific time interval between AP episodes [8]. We enrolled 130 adult patients with first AP attack from the medical record database as the control group using the random number table method. The criteria for adult AP were in accordance with the Atlanta International consensus 2012 [9]. We compared the clinical characteristics between children with AP (child group) and adults with AP (adult group). In order to analysis of the clinical characteristics of AP in children of different ages, patients were divided into two groups: younger and older children group (< 10 years and ≥ 10 years). Based on the follow-up results, we divided 130 children patients into recurrence groups and the non-recurrence group, and analyzed the risk factors associated with ARP in children.

### Clinical data collection

Data were collected by researchers who were not informed of the detailed information of the study until collection was completed. The collected data were from computer-based case reports and were checked by another two researchers to ensure that there were no inconsistencies or errors. Basic information on these patients was collected, including age, sex, etiologies, clinical symptoms, laboratory indexes within 24 h of admission (blood amylase, lipase, triglyceride (TG), C-reactive protein (CRP)), imaging findings, complications, and treatment outcome. We retrieved the data of the second AP attack hospitalization in the database for 18 of the 130 children. They were labeled as ARP. We conducted telephone follow-up of 112 patients, an 8-years-old child with traumatic AP was diagnosed with RAP in another hospital due to abdominal pain after the first attack AP discharge 3 months, and the remaining 111 patients without abnormalities. The follow-up period was 1 to 72 months, with a mean time of 34.2 ± 20.8 months, and the median time of follow-up was 32.5 (IQR 16–50.3) months.

### Statistical analysis

The data were analyzed using SPSS software version 25.0 (IBM Corporation, Armonk, NY, USA). Continuous variables were described by mean ± standard deviation or median (range) and classified variables by percentages. Comparison of non-normally distributed data and hierarchical data groups used the Mann–Whitney U test. The chi-squared test and Fisher’s exact probability test were employed to complete the univariate analysis. The chi-squared test for trend was used for unidirectional ordered classification, and Goodman–Kruskal gamma analysis was used for variables with bidirectional ordered classification. Univariate and multivariate Cox regression were used determine adjusted odds ratios. A Cox proportional hazards model was used to analyze etiology groups and time to ARP for the whole sample. The cumulative recurrence curve used Kaplan–Meier method through R software version 3.6.0 (https://www.r-project.org) and Log-rank test was used for comparison between groups. The software packages we used during the analysis include survival and survminer. *p* < 0.05 was considered statistically significant for analyses.

## Results

### Baseline characteristics

Major etiologic factors in 130 patients were biliary (31.5%)(including 29 cases of bile duct stones, 8 cases of congenital biliary dilatation, 4 cases of biliary tapeworm) and a significant proportion (28.5%) of cases no cause could be found, we label them as idiopathic. In addition, definite causes include trauma (16.2%), viral infection (10%), hyperlipidemia (9.3%)(TG > 11.30 mmol/L in 12 children), and drug-induced (4.6%)(including 5 children were taking dexamethasone and 1 child with leukemia was receiving chemotherapy with Cytarabine). There was a significant difference in the etiological constituent ratio between children and adults (*p* < 0.001): cases in children were mainly biliary and idiopathic, and those in adults were mainly biliary and hypertriglyceridemia. The severity of AP in the children was milder than that in the adult group (*p* = 0.018), and the rate of ANC, APFC, SIRS in the children was lower than that in the adult group (all *p* < 0.05).

### Clinical characteristics based on age

AP was more likely to occur in older age groups of children. However, the severity of the disease was independent of the increase in age, and there was no significant difference in sex. There were significant differences in the etiology of different age groups, viral infectious pancreatitis was more likely to occur in younger children, and the older children were usually due to biliary and hypertriglyceridemia (*p* = 0.013). APFC was more likely to occur in older children (*p* = 0.024).

### Treatment

All patients admitted were given standard medical treatment. Patients received treatment including fasting, early fluid resuscitation, analgesia, and nutritional support. 123 of 130 patients were cured and discharged, 7 patients were improved or discharged for further treatment. 8 of 130 children patients were treated with cholecystectomy, a child was treated with endoscopic retrograde cholangiopancreatography (ERCP). 14 of 130 children patients had pancreatic pseudocysts, 10 patients of which healed after medical treatment, 4 patients underwent pseudocyst drainage.

### Risk factors for ARP

Of the 130 children with AP, 19 (14.6%) progressed to ARP during the study period. The mean interval from AP to ARP was 9.5 ± 6.9 months, and the mean age at the second AP attack was 12.1 ± 4.2 years. The recurrence rate increased with an increase in disease severity (*p* = 0.034). Female sex (*p* = 0.001), hypertriglyceridemia (*p* = 0.016), APFC (*p* = 0.044), and the presence of ANC during the first AP attack (*p* = 0.040) were significantly correlated with ARP (Table 1).

**Table 1:** Characteristics of patients in recurrence and non-recurrence groups

Variables	Recurrence group	Non-recurrence group	*p* Value
	n = 19	n = 111	
Age, years	11.2 ± 4.2	11.3 ± 4.2	0.926^a^
Sex, n%			0.001^b^
Male	4 (5.6%)	68 (94.4%)	
Female	15 (25.9%)	43 (74.1%)	
Etiology, n%			
Biliary	5 (12.2%)	36 (87.8%)	0.804^b^
Idiopathic	6 (16.2%)	31 (83.8%)	0.745^b^
Trauma	3 (14.3%)	18 (85.7%)	0.963^b^
Hypertriglyceridemia	5 (41.7%)	7 (58.3%)	0.016^c^
Viral infection	0 (0%)	13 (100%)	0.116^b^
Drug-induced	0 (0%)	6 (100%)	0.299^b^
Severity, n%			0.034^d^
MAP	11 (11.5%)	85 (88.5%)	
MASP	6 (20%)	24 (80%)	
SAP	2 (50%)	2 (50%)	
Complication, n%			
APFC	7 (36.8%)	16 (14.4%)	0.044^c^
ANC	3 (15.8%)	3 (2.7%)	0.040^c^
PPC	2 (14.3%)	12 (85.7%)	1.000^c^
SIRS	4 (21.2%)	8 (7.2%)	0.075^c^

^a^Mann-Whitney U test

^b^Chi-square test

^c^Fisher’s exact test

^d^Trend Chi-squared test

MAP: mild acute pancreatitis, MASP: moderately-severe acute pancreatitis, SAP: severe acute pancreatitis, APFC: acute peripancreatic fluid collection, ANC: acute necrotic collection, PPC: pancreatic pseudocyst, SIRS: systemic inflammatory response syndrome

COX regression analysis for the above meaningful indicators. Univariate analysis and multivariate Cox regression analysis showed that female sex (*p* = 0.020; OR = 3.821; 95%CI 1.231–11.861), hypertriglyceridemia (*p* = 0.045; OR = 3.111; 95%CI 1.024–9.447), and pancreatic necrosis (*p* = 0.023; OR = 5.768; 95%CI 1.278–26.034) were the independent factors influencing ARP (Table 2).

**Table 2:** Univariate and Multivariate Cox regression analysis of factors associated with ARP in children

Variable	Univariate analysis		Multivariate analysis	
	OR (95%CI)	*p* Value	OR (95%CI)	*p* Value
Age	0.999 (0.899–1.111)	0.992	–	–
Sex, Female	4.626 (1.535–13.941)	0.007	3.821 (1.231–11.861)	0.020
Hypertriglyceridemia	4.505 (1.619–12.537)	0.004	3.111 (1.024–9.447)	0.045
APFC	3.049 (1.200–7.751)	0.019	1.462 (0.476–4.492)	0.507
ANC	5.792 (1.678–19.996)	0.005	5.768 (1.278–26.034)	0.023
PPC	0.970 (0.224–4.200)	0.968	–	–
SIRS	2.837 (0.941–8.553)	0.064	–	–

MAP: mild acute pancreatitis, MASP: moderately-severe acute pancreatitis, SAP: severe acute pancreatitis, APFC: acute peripancreatic fluid collection, ANC: acute necrotic collection, PPC: pancreatic pseudocyst, SIRS: systemic inflammatory response syndrome

**Fig. 1:**
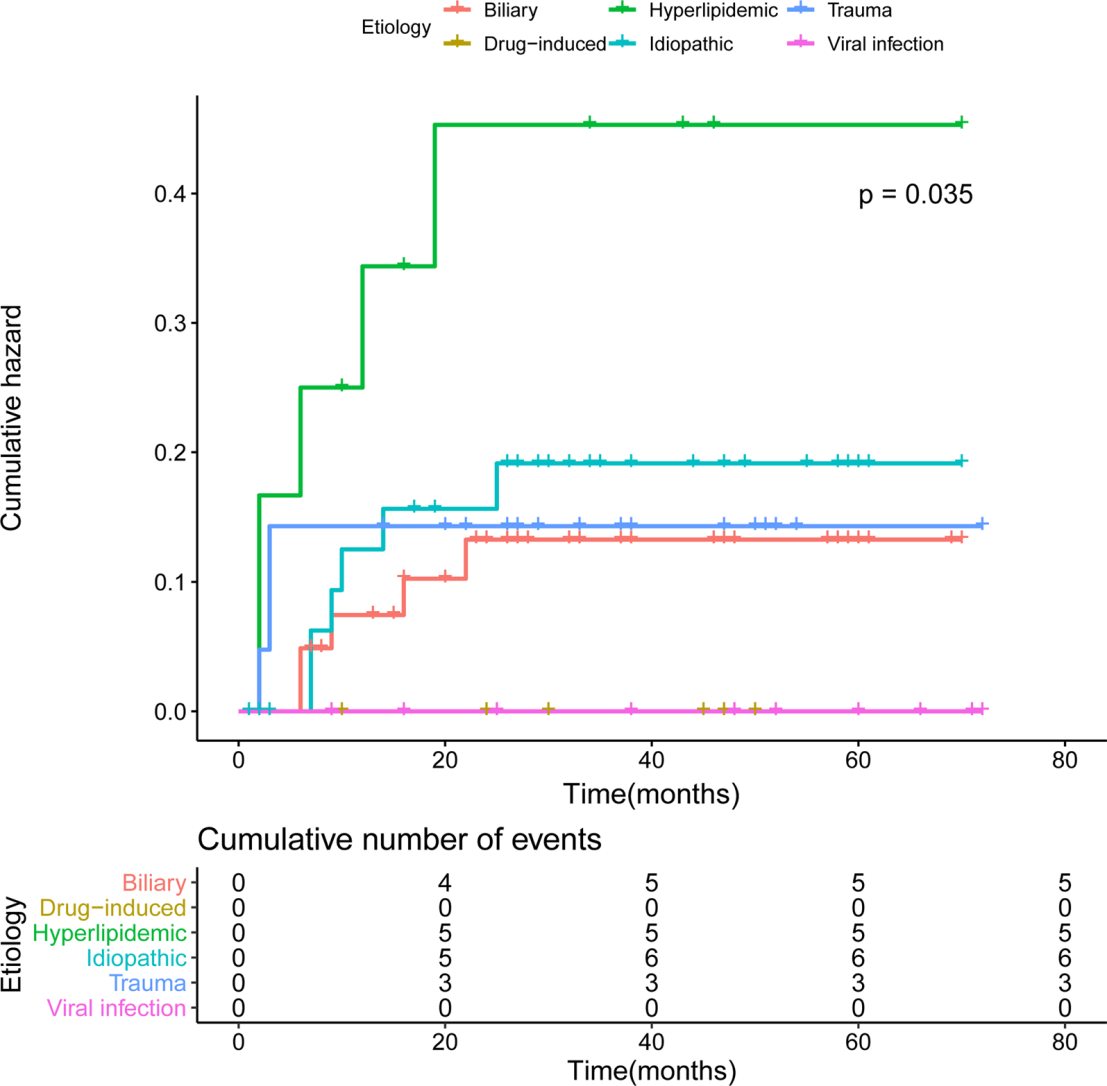
Risk of acute recurrent pancreatitis (ARP) by etiology

We were also interested in studying the etiology or risk factors associated with the first AP occurrence and their effect on progression to ARP over time for the whole sample. We compared patients with biliary, idiopathic, traumatic, hypertriglyceridemia, viral, and drug-induced AP. We found that hypertriglyceridemia AP had the highest risk of recurrence over time, while viral and drug-induced AP had the lowest risk of recurrence (*p* = 0.035). (Fig. 1).

## Discussion

In the past ten years, the epidemiology and etiology of pediatric pancreatitis has been greatly developed [10–12]. But AP is uncommon in children, and the limited number of examined children in the available, current literature, it seems necessary to plan a multi-center study in order to evaluate the incidence, etiology, natural history, and evaluation of the related risk factors [13]. In this study, the clinical characteristics of AP in children in southwest China were analyzed and summarized. As far as we know, this is the first report in English on the clinical features of AP in children from China.

The etiology of AP in children was significantly different from that in adults, as was found in this cohort. In previous studies, Poddar et al. [14] studied 320 children with AP from India and found that trauma (21%) and biliary tract disease (10%) were the most common causes of AP in children. Park et al. [15] found that the biliary tract (36.2%) and drugs (25.6%) were the leading causes of AP in 215 children in the United States. In another study of 115 children in the United States [16], idiopathic (31%) and drug associated (23%) were the main causes. We found that biliary (31.5%), idiopathic (28.5%) and traumatic (16.2%) were the main causes, which was consistent with most studies. In addition, metabolic problems such as hypertriglyceridemia in children were significantly rarer than in adults. Only 2–7% of children with AP have metabolic causative factors [10, 11]. In this study, 12 cases (9.2%) were caused by hypertriglyceridemia. In the control group of adults, high fat levels accounted for 26.2%, and the difference was significant. The most exciting finding in our study was that viral infectious pancreatitis was more common in young children and the rate was significantly different from that in adults. We consider that is was related to the incomplete development of the immune system in young children. The mechanism of AP related to virus infection is not clear at present, although some research has shown that the virus directly invades pancreatic cells [3]. Although drug-induced pancreatitis has increased significantly in recent years with the widespread use of drugs, it is still constitutes less than 10% of cases [17].

Our study showed that the severity of AP in children was similar in all age groups, which was consistent with a previous report [3], but the disease was less severe than in adults. Possible reasons are: (1) In terms of etiological composition, hyperlipidemia and alcoholic pancreatitis account for few cases in children, and some studies have shown that hyperlipidemia and alcohol can easily lead to SAP [18]; (2) In terms of complications, pancreatic necrosis was positively correlated with the severity and prognosis of the disease. Necrotizing pancreatitis was rare in children. According to the literature [19], necrotizing pancreatitis occurs in less than 1% of children with AP. Among the five large sample pediatric cases reported in the United States [20], only 3 of 1014 children with AP had pancreatic necrosis, which was significantly less severe than that of adults. Our research results also confirmed the above view.

For AP with specific causes, such as bile duct stones, anatomical abnormalities, bile duct dilatation, etc., it has been suggested that surgery should be performed as soon as possible after the condition is controlled and stable, to prevent recurrence [11, 21]. No child died in our research data. In other literature [22], the mortality rate of pediatric AP is less than 5%, which is significantly lower than that of adult AP, possibly because: (1) Alcoholic pancreatitis is rare in pediatric cases, and alcoholic pancreatitis is a known cause of high mortality, with a mortality rate as high as 30.6% [23]. (2) With age, adults may lose some critical protective mechanism, which children retain [6]. (3) Adult cases may be associated with severe underlying diseases, while AP in children is rarely associated with multiple problems with organ function. (4) The severity of the disease in children is significantly lower than that in adults, which has also been confirmed in this study.

Regarding ARP in children, some single-center studies have estimated that 10–35% of children with AP develop ARP [24, 25]. The main driving force behind the progression of acute pancreatitis to recurrent acute and chronic pancreatitis is genetic predisposition [26]. These studies showed that mutations of PRSS1, SPINK1, CFTR, and CTRC13 were firmly related to the progression of ARP. During the first attack of AP in children, age, male sex, pancreatic necrosis, and higher Body Mass Index (BMI) have been associated with the progression of ARP, anatomic abnormalities of the biliary tract, hyperlipidemia, and genetic factors should be evaluated in cases of recurrence [11, 16, 27]. We found that female sex, hypertriglyceridemia, and primary AP with pancreatic necrosis were significantly correlated with ARP, and the severity of primary AP was positively correlated with ARP. The relationship between ARP and sex is controversial and perhaps related to the predominance of hyperlipidemia and biliary tract disease in women [28, 29]. Differences in our findings from those of from previous studies may have resulted from differences in race, region, and environment. Hyperlipidemia is an apparent cause of AP and ARP in adults [30], but there has been no analysis of a large sample of ARP cases in children. It has been reported in the literature that hypertriglyceridemia occurring secondary to AP may be related to various genetic mutations [31]. From this point of view, our findings coincide with previous research results.

The disadvantage of this study was that it was a single-center study. Thus, the findings of this study cannot be generalized. For the 19 patients with ARP, the etiology of recurrence still needs to be examined by ERCP and genetic analysis, and there is a lack of corresponding genetic and clinical data in this study. In future, multi-center studies with a larger sample and more rational perspective are needed to analyze pediatric pancreatitis in order to facilitate better treatment.

## Conclusion

Biliary disease and idiopathic cases were the leading causes of AP in children, and, when compared with adults, children tend to have milder disease and a better prognosis. The recurrence rate increased with an increase in disease severity. Female sex, hyperlipidemia, and a first AP attack involving pancreatic necrosis were associated with an increased risk of ARP. Genetic and anatomical factors need to be studied further in children with ARP.

## Data Availability

All data generated or analyzed during this study are included in this published article.

## Abbreviations

AP: Acute pancreatitis
APFC: Acute peripancreatic fluid collection
ARDS: Acute respiratory distress syndrome
ARF: Acute renal failure
ARP: Acute recurrent pancreatitis
BMI: Body Mass Index
CRP: C-reactive protein
ERCP: Endoscopic retrograde cholangiopancreatography
IQR: Interquartile range
MAP: Mild acute pancreatitis
MASP: Moderately-severe acute pancreatitis
SAP: Severe acute pancreatitis
SIRS: Systemic inflammatory response syndrome
TG: Triglyceride
